# Simultaneous evoked CMAP recording and sonography reveal a prolonged electromechanical delay in ALS patients

**DOI:** 10.3389/fneur.2026.1943566

**Published:** 2026-10-28

**Authors:** Sebastian A. Wölk, Uwe Walter, Johannes Prudlo, Anne-Kathrin Ruß, Robert Patejdl

**Affiliations:** 1 Oscar Langendorff Institute of Physiology, Rostock University Medical Center, Rostock, Germany; 2 Department of Neurology, Rostock University Medical Center, Rostock, Germany; 3 German Center for Neurodegenerative Diseases (DZNE) Rostock/Greifswald, Rostock, Germany; 4 Department of Medicine, Health and Medical University Erfurt, Erfurt, Germany

**Keywords:** amyotrophic lateral sclerosis, distal motor latency, electrodiagnosis, electromechanical coupling, electromechanical delay, mechanical latency, muscle ultrasound, sonoelectromyography

## Abstract

Alterations in electromechanical coupling have been described in patients with amyotrophic lateral sclerosis (ALS) and may contribute to the pathophysiology of the disease. Regardless of this, few measurements of electromechanical delay (EMD) in ALS patients have been published. In this motor nerve conduction study, 28 patients with ALS were examined for changes in electromechanical coupling time compared to healthy individuals using sonoelectromyography, a novel dual-method approach combining simultaneous evoked compound muscle action potential (CMAP) recording following an ENG protocol with Doppler ultrasound. In the examination of four muscles (the abductor digiti minimi muscle, brachioradialis muscle, tibialis anterior muscle, and rectus femoris muscle) ALS patients exhibited prolonged electromechanical delay times. These results demonstrate alterations in the electromechanical properties of muscle fibers and provide further insight into muscle contractility in ALS. The findings support the advancement and wider utilisation of sonoelectromyography as an innovative tool for assessing muscle function and indicate that EMD could be incorporated into diagnostic and disease monitoring protocols in ALS.

## 1 Introduction

Amyotrophic lateral sclerosis (ALS) is a progressive neurodegenerative disease. Motor neuron degeneration in the spinal cord, brainstem, and cortex is a central pathological feature (1). The progressive destruction of motor neurons can lead to muscle weakness, muscle atrophy, dysphagia, and dysarthria (2). The effects of the disease are not solely limited to visible muscle atrophies and paralysis. In the skeletal muscles of ALS patients, complex functional modifications are identified that go beyond the typical neurogenic lesion pattern, involving the degeneration of the neuromuscular junction and alterations in the contractile properties of muscle fibers (3, 4).

Throughout the disease progression, neuronal plasticity facilitates axonal sprouting and re-innervation (5, 6). Despite these adaptive processes, the original state is not fully reinstated. Subsequent to these alterations, motor units in ALS patients demonstrate enlargement (7), as evidenced by enlarged motor neuron action potentials. Mechanically and myoelectrically, these enlarged motor units exhibit functional variability (4). During the course of the disease, contractions become mechanically less efficient, resulting in reduced force generation alongside quicker fatigue (8). Dysfunctions in electromechanical coupling appear to contribute to this phenomenon (4, 9).

Muscle structure and function analyses using B-mode ultrasound imaging have become widely accepted in clinical practice. The consistent detection of fasciculations has led to a growing interest in incorporating muscle ultrasound as a standard diagnostic approach in the diagnosis of ALS (10, 11). Further research is required to standardize muscle ultrasound for ALS diagnosis and monitoring (12).

Muscle function assessments using Doppler ultrasound have not yet been widely established, but findings from individual studies suggest that it may be worthwhile to pursue this avenue further (13).

This study aims to investigate contractile muscle properties in ALS patients in comparison to healthy controls. In particular, changes in electromechanical coupling are to be characterized using a novel method that combines surface electromyography with sonographic muscle motion analysis. Electromechanical coupling is assessed in individual muscles by analyzing the temporal relations between externally evoked compound muscle action potentials (CMAP) and contraction-related Doppler-signals derived from the same muscle.

## 2 Materials and methods

### 2.1 Principles of the method

The analysis of muscle activity using Doppler ultrasound relies on the presence of distinct acoustic compartments with different acoustic impedances, which are provided in skeletal muscle by connective tissue layers surrounding muscle fibres and fibre bundles. These interfaces act as acoustic scatterers and reflect part of the incident ultrasound wave back towards the transducer. When these scattering structures move relative to the ultrasound transducer, the frequency of the returning ultrasound differs from that of the transmitted wave. This phenomenon, known as the Doppler effect, enables the movement of tissue structures to be characterized through the resulting Doppler frequency shift (14). The magnitude of this shift is related to the velocity component of tissue movement along the ultrasound beam, with faster tissue movement producing a larger frequency shift and slower movement producing a smaller shift. Movement towards the transducer results in a positive frequency shift, while movement away from the transducer produces a negative shift. During muscle contraction, the muscle fibres undergo both longitudinal shortening and transverse thickening, thereby changing the position of tissue interfaces and scattering structures relative to the ultrasound beam. In particular, transverse thickening during contraction results in movement of tissue structures towards the Doppler probe, whereas relaxation is associated with movement away from the probe. The resulting temporal changes in the Doppler frequency shift therefore reflect the changing velocity and direction of tissue movement during muscle activation and relaxation. The effect of muscle thickening during contraction on the Doppler frequency is shown schematically in Figure 1. Low-frequency Doppler components arising from slow tissue movements can be suppressed by means of a wall filter, thereby allowing the higher-frequency components associated with more rapid tissue movement to be distinguished. Consequently, the temporal variation in the Doppler frequency shift provides information about the velocity of muscle tissue movement and gives rise to the characteristic waveform recorded by the ultrasound probe (47).

**Figure 1:**
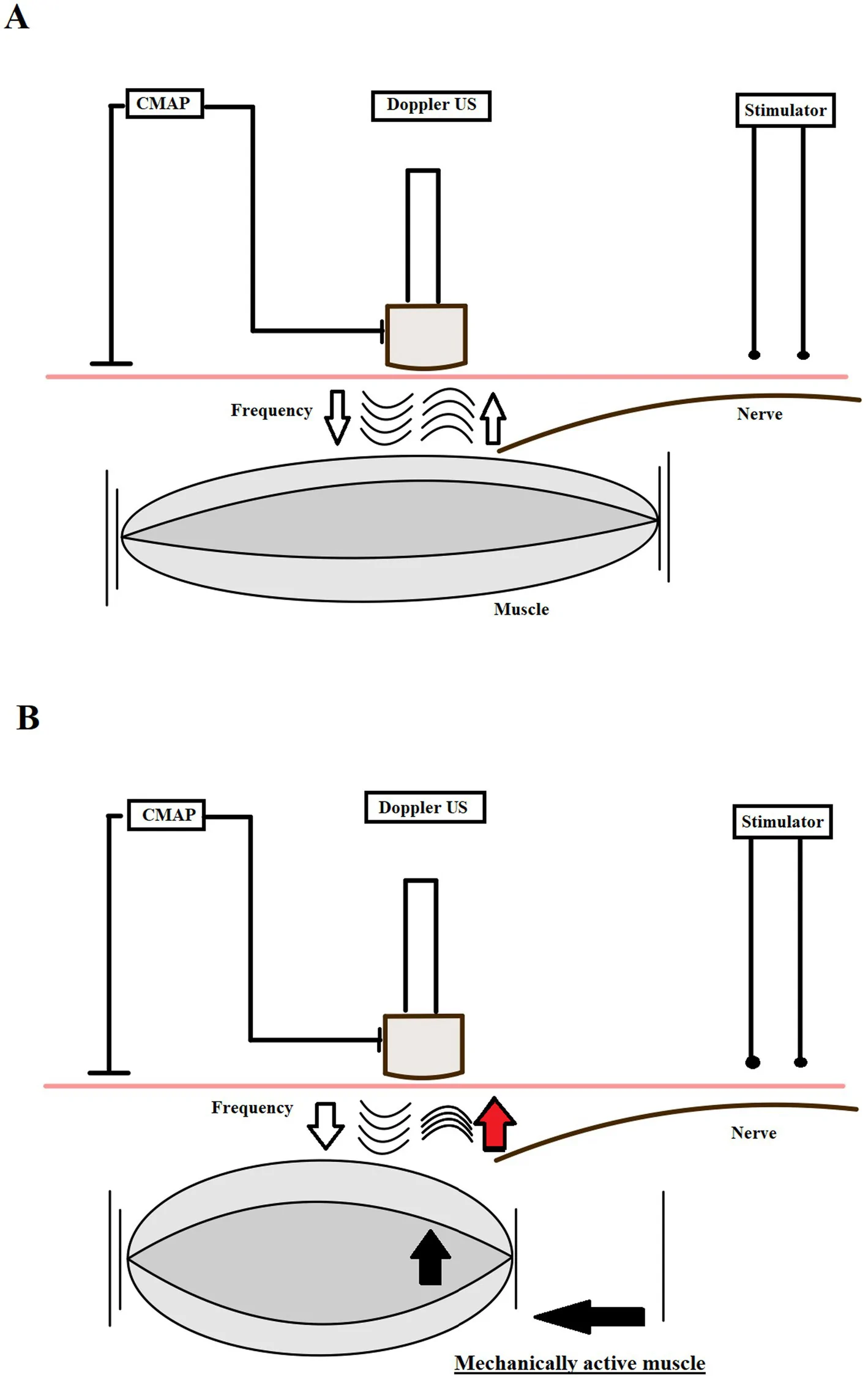
Experimental setup and schematic representation of the Doppler effect on a resting **(A)** and a mechanically active **(B)** muscle. During contraction, muscle thickening moves the tissue toward the probe, causing a positive Doppler shift.

### 2.2 Subjects

The results of the ALS cohort were compared with two healthy control groups: an age-matched group and a control cohort of younger individuals. Demographic and disease-relevant factors are presented in Table 1. A total of 28 individuals were included in the ALS group. Of these, 25 met the criteria for classic ALS, characterized by combined upper and lower motor neuron involvement (15). Three of these patients presented with progressive bulbar palsy. The remaining three individuals met the diagnostic criteria for primary lateral sclerosis (PLS). Patients were variably affected, reflecting differences in disease progression and the extent of upper and lower motor neuron involvement. The ALS patients had a mean ALSFRS-R score of 34 ± 8 and a median disease duration of 16 months (IQR, 12.25 months). Muscle strength was assessed using the MRC scale for elbow flexion, wrist extension, knee extension, and ankle dorsiflexion. Considering the weaker side for each muscle group, the combined MRC muscle strength score for these four muscles was 16.6 ± 3.1 points out of a maximum of 20. Alongside demographic variables, additional disease-specific clinical parameters were recorded for all ALS patients. These included the presence of fasciculations, reflex status, degree of spasticity, and the degree of muscle atrophy in the extremities.

**Table 1:** Demographics and characteristics of the examined groups.

Characteristics	ALS	Age-matched controls	*p*	Younger controls	*p*
Sex (M:F)	16:12	16:13	1 Fi.	16:9	0.78 Fi.
Age (years)	66.0 ± 9.7	70.2 ± 9.1	0.103 t	24.1 (4)	<0.001 U
Body height (m)	1.72 ± 0.1	1.71 ± 0.1	0.703 t	1.77 ± 0.2	0.041 t
Weight (kg)	73 ± 12	82 ± 21	0.153 U	70.4 ± 11	0.389 t
BMI (kg/m^2^)	24.7 ± 3.1	28.2 (5.5)	0.028 U	22.4 ± 2.3	0.003 t
Cramp index score	65 (48)	4 (3)	<0.001 U	0.2 (0)	<0.001 U
ALSFRS-R	34 ± 8 35 (11.25)				
Disease duration (months)	16 (12.25)				
Onset region (bulb:C, Th, L/S)	9:19				
Modified Ashworth score	1 ± 1.3				
Combined MRC muscle strength score (0–20)	16.6 ± 3.1				
At least mild atrophies in:					
Arms (Yes:No)	20:8				
Legs (Yes:No)	14:14				
At least mild fasciculations in:					
Arms (Yes:No)	12:16				
Legs (Yes:No)	13:15				
Lively or heightened reflexes in:					
Arms (Yes:No)	18:10				
Legs (Yes:No)	21:7				

bulb = bulbar, C = cervical, Th = thoracic, L/S = lumbosacral, modified Ashworth score ranging from Grade 0 (no increased tone) to Grade 4 (significantly increased tone) (46), Cramp Index score = number of cramps per month × severity (1–4) × duration (1–3) (23). The combined MRC muscle strength score was calculated by summing the MRC grades (0–5) of the weaker side for elbow flexion, wrist extension, knee extension, and ankle dorsiflexion, resulting in a maximum score of 20. The *p*-values indicate statistical differences between the ALS cohort and either age-matched or younger controls, as determined by Fisher’s exact test (Fi.), the *t*-test (*t*), and the Mann–Whitney *U* test (*U*). The value in parentheses represents the interquartile range in cases where the group does not follow a normal distribution.

For the first control group (age-matched controls), 29 subjects of similar age and gender without motor neuron disease were recruited. The control group primarily consisted of healthy volunteers, with one-third of the participants being individuals hospitalized for a transient ischemic attack who showed no signs of motor neuron disease or clinically manifest polyneuropathy. A mandatory inclusion criterion for these participants was the objective absence of central structural neurological lesions on cerebral MRI, including T1, T2, and two-plane diffusion-weighted sequences. Regarding the peripheral nervous system, a strict inclusion requirement was the complete absence of any clinical signs of peripheral neuropathy, radiculopathy, or focal nerve lesions, as verified by a clinical neurological examination demonstrating completely normal muscle strength, normal deep tendon reflexes, and intact sensory function in all examined limbs. The second control group (younger controls) consisted of 27 healthy subjects aged between 21 and 29 years.

This dual-control approach was implemented to establish a broad, comprehensive normative baseline across different age spans. By incorporating a second control cohort, we aimed to maximize the total number of healthy control data points within the linear mixed model, thereby increasing statistical power and ensuring a highly stable normative baseline. Furthermore, evaluating this second control cohort allowed us to systematically verify whether electromechanical delay measurements remain stable and independent of chronological aging in healthy subjects, thereby validating the method as a robust, disease-specific marker.

The inclusion of subjects or patients occurred after thorough explanation and obtaining consent from the individual or their legal representative. All explanations were documented in writing.

### 2.3 Experimental procedure

The experimental setup followed a conventional motor nerve conduction study protocol. A peripheral nerve was stimulated supramaximally, and the signal was derived through active, reference, and ground electrodes. In addition to recording the CMAP, the mechanical properties of muscle activity were simultaneously assessed using Doppler measurements. CMAP amplitudes were quantified using the peak-to-peak method. To establish accurate correlations between electrical and mechanical signals, both signals had to be acquired from the same anatomical site. This approach enabled the determination of temporal delays between electrical and mechanical events. To achieve this, the active electrode was constructed as a ring electrode, allowing for the insertion of a Doppler probe (Figure 2). Consequently, Doppler measurements and electrical recordings could be conducted concurrently at a distinct anatomical site. A depiction of the measurement setup is illustrated in Figure 1.

**Figure 2:**
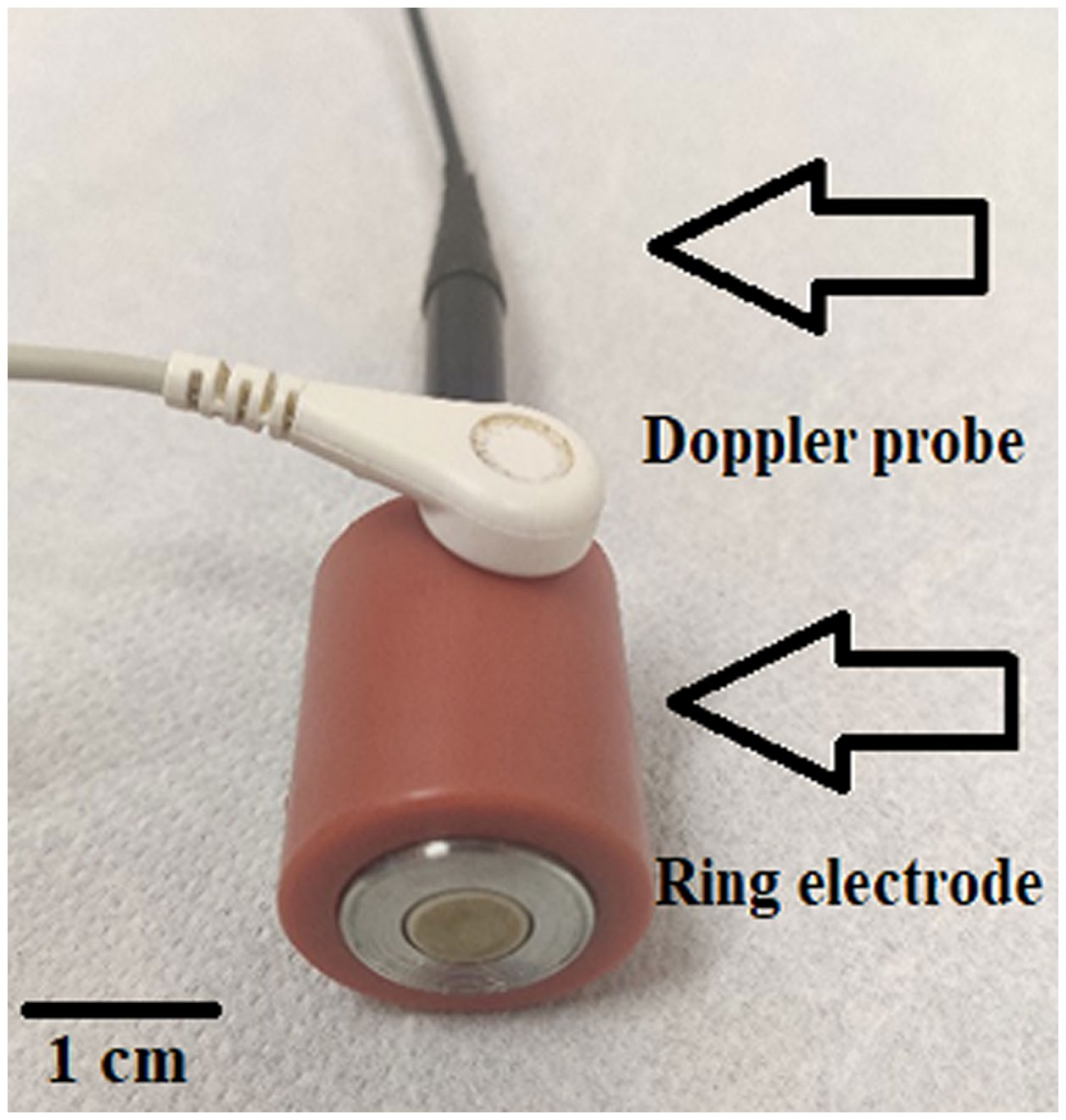
Measurement setup with a Doppler probe and an active ring electrode. The specific measurement setup allows for simultaneous measurement of electrical and mechanical signals of the muscle.

To investigate electromechanical coupling in ALS patients, two muscles of the upper extremities and two muscles of the lower extremities were examined bilaterally. For the arm, the abductor digiti minimi muscle (ADM) and the brachioradialis muscle (BR) were selected, while for the leg, the tibialis anterior muscle (TA) and the rectus femoris muscle (RF) were chosen. The positions of the recording electrodes were based on clinical standards (16). For the ADM, the active electrode was placed halfway between the pisiform bone and the fifth metacarpophalangeal joint. The reference electrode was positioned slightly distal to the metacarpophalangeal joint. For stimulation, the cathode of a bar electrode was placed eight centimeters proximal to the active electrode on the ulnar nerve. The experimental setup for the ADM is schematically shown in Figure 3. When measuring the BR, the radial nerve was stimulated five centimeters proximal to the lateral epicondyle of the humerus, with the active electrode positioned three centimeters distal to the bony prominence and the reference electrode placed slightly distal to the thumb metacarpophalangeal joint. For the TA, stimulation was applied at the fibular head to the deep fibular nerve, with the active electrode positioned eight centimeters distal to this point and the reference electrode placed on the ventral distal tibia. For the RF, the femoral nerve was stimulated below the inguinal ligament, with the active electrode positioned 15 centimeters distal to this point and the reference electrode placed slightly below the patella. The stimulation intensity was chosen to achieve supramaximal contraction. All measurements were performed with the participant in a relaxed supine position, with a knee roll placed under the knees. The joints were not mechanically fixed, allowing for movement following stimulation, and the exact limb positioning was not strictly standardized across participants.

**Figure 3:**
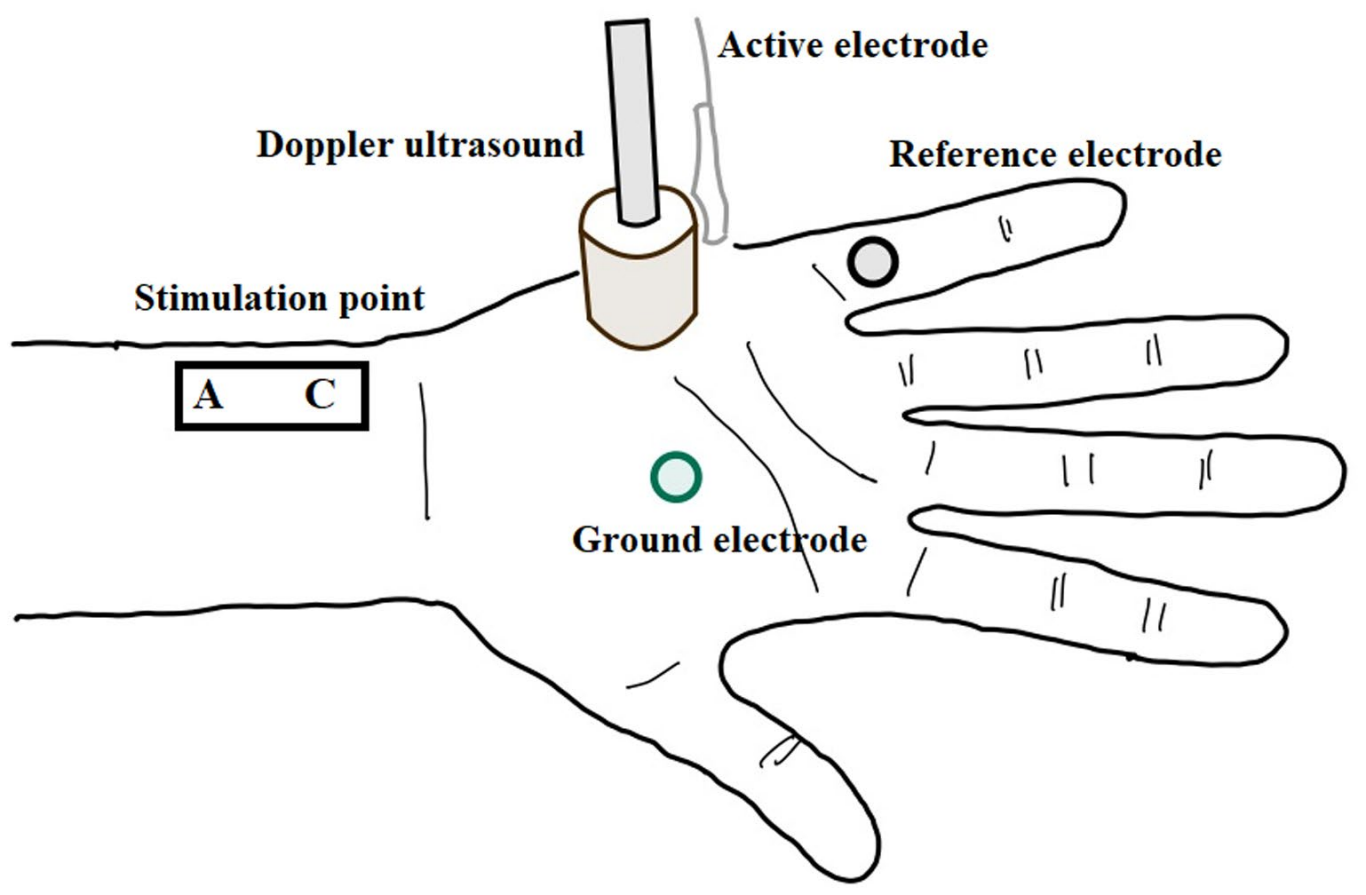
Schematic measurement setup for the ADM. The measurement setup utilizes a steel ring electrode to enable simultaneous Doppler signal recording at the active electrode location.

Figure 4 depicts a segment of the stimulation measurement for the abductor digiti minimi muscle. The distal motor latency (DML) is measured from the onset of the electrical stimulation recording to the initial deflection of the evoked CMAP response. The interval to the first deflection of the Doppler curve is referred to as mechanical latency. The electromechanical delay (EMD) is defined as the period from CMAP onset to the first mechanical deflection and is calculated as EMD = mechanical latency − DML.

**Figure 4:**
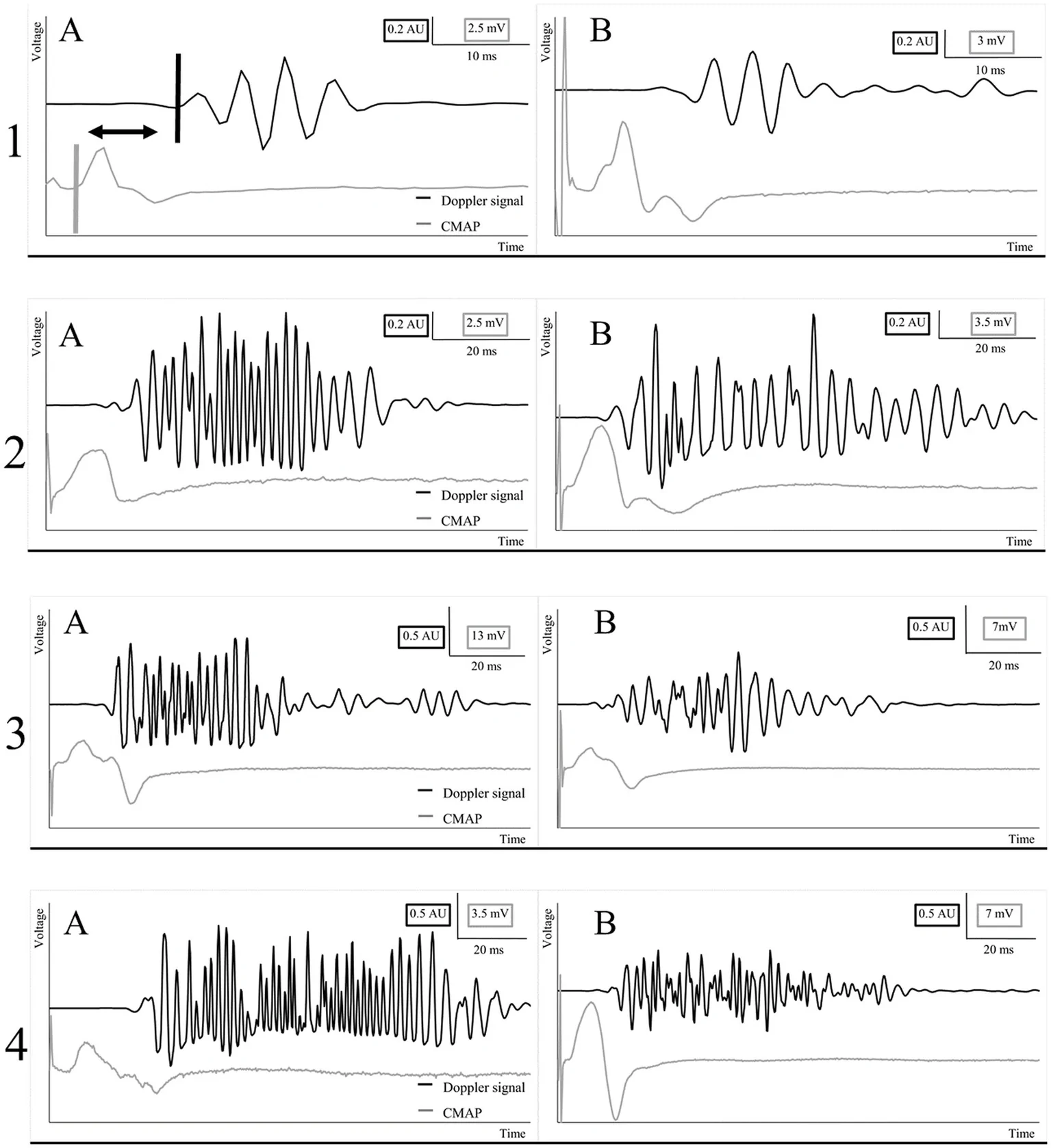
Exemplary measurement recordings of Doppler and CMAP signals for ALS patient No. 27 **(A)** and age-matched control subject No. 15 **(B)** across the abductor digiti minimi (1), brachioradialis (2), tibialis anterior (3), and rectus femoris (4) muscles. Panel **(1A)** shows an exemplary measurement of the electromechanical delay. The gray vertical line indicates the measurement point for the distal motor latency, while the black horizontal bar indicates the measurement point for the mechanical latency. Recordings are displayed using muscle-specific dual Y-axis configurations, illustrating the Doppler signal in black on the left left Y-axis in arbitrary units [au] and the CMAP amplitude in grey on the right Y-axis in millivolts [mV] against a synchronized time scale, where the size of the time interval is indicated by the length of the horizontal scale bar. The electromechanical delays for the abductor digiti minimi (1), brachioradialis (2), tibialis anterior (3), and rectus femoris (4) muscles were 5.55 ms, 8.75 ms, 9.75 ms, and 15.50 ms for the ALS patient, compared to 4.25 ms, 6.00 ms, 7.25 ms, and 9.63 ms for the control subject, respectively.

Stimulation was conducted using a Keypoint Dantec device (Keypoint Dantec; Natus Medical Incorporated, Planegg, Germany) with a biphasic pulse width of 200 μs. A conventional Doppler device with an 8 MHz Probe was used in continuous wave—mode (SonoPan UDP10, Poland). Bioelectrical and acoustic signals were recorded with a PowerLab system (PL2604; ADInstruments, Oxford, United Kingdom) and transferred to a LabChart program (LabChart version 7; ADInstruments, Oxford, United Kingdom) for analysis.

### 2.4 Signal processing and statistical analysis

The continuously generated Doppler signals were directly routed to one input channel of the four-channel PowerLab data acquisition system, while the electrical CMAP signal was simultaneously fed into another dedicated input channel. Recording synchronization was initiated by coupling the transistor–transistor logic (TTL) stimulation output directly to the external trigger input of the PowerLab hardware, thereby ensuring that the Doppler probe and the CMAP recording were strictly synchronized.

Onset latencies, defined as the first clear baseline deviation preceding the peak, were manually verified via visual inspection. This single-investigator approach was chosen to bypass filter artifacts and guarantee consistent measurement. We utilized visual inspection because it is widely recognized as the gold standard for its high precision in capturing onset markers in electrophysiological signals (17).

The LabChart measurement parameters comprised a sampling rate of 40 kHz, an input range of 50 mV for CMAP recording, and a Doppler input range of 2 V. Low- and high-pass filters were set at 10 Hz and 1 kHz, respectively, and a mains filter was applied during signal acquisition.

The Kolmogorov–Smirnov test was employed to evaluate the normal distribution of the dataset. For comparing means of two groups, the students *t*-test was primarily conducted. If there was no normal distribution or equal variance, the comparison was performed using the Mann–Whitney Rank Sum Test. Unless otherwise specified, the values of the ALS cohort were compared with those of age-matched or younger controls. Statistical significance was defined at *p* < 0.05. All statistical tests were two-sided. The data are primarily presented as the mean ± standard deviation. When the data do not follow a normal distribution, the interquartile range is provided in parentheses. The 95% confidence interval of the mean is shown in square brackets. Spearman’s rank correlation coefficients were interpreted based on their strength: ≥0.8 was considered very strong, 0.6–0.8 moderately strong, 0.3–0.5 fair, and less than 0.3 poor (18). For a standardized analysis across subgroups, raw data were normalized using z-score transformation. These tests were performed using SigmaStat (version 3.56, Systat Software GmbH, Erkrath, Germany), IBM SPSS Statistics (version 29, IBM Corp., Armonk, NY, USA), and the Data Analysis ToolPak in Microsoft Excel (Microsoft Corp., Redmond, WA, USA).

To account for the repeated measurements, a linear mixed-effects model (LMM) was additionally employed using R (19) version 4.6.1 (2026-06-24 ucrt) on the x86_64-w64-mingw32/x64 platform. This LMM was fitted using the lmer function from the lme4 package (20). For each patient, measurements were taken on both sides of the body at various muscles.

In this model, EDM was outcome, age, sex, group (ALS, matched controls and younger controls) and muscle (ADM, BR, RF and TA) were included as fixed effects, whereas patient was treated as random effect to account for measurement repetitions. As for each muscle both sides, left and right, were measured, we checked for side effects applying different modelling approaches. No significant effects were observed here; therefore, we did not take the side of the body into account in the presented results.

Examination of the model residuals revealed considerable deviations from the normal distribution. Therefore, we log-transformed the outcome which resulted in normally distributed values. To determine overall *p*-values for nominal variables, we compared the full model with the model excluding the respective variable. Pairwise comparisons of the categories were conducted post-hoc using the emmeans package (21). These *p*-values were calculated using the Wald test, incorporating Satterthwaite degrees of freedom and a Tukey correction for multiple testing.

In order to evaluate the potential clinical relevance of the collected parameters, their diagnostic efficacy was assessed utilizing Receiver Operating Characteristic (ROC) analysis, which juxtaposes sensitivity and 1-specificity. The determination of cutoff values was primarily conducted employing the Youden Index, a metric of discrimination ranging from 0 to 1 (22). Additionally, the area under the curve (AUC) was calculated as a criterion for test performance evaluation.

When the full stimulation protocol could not be completed or was prematurely discontinued by a participant, all valid partial measurements were included in the analysis.

## 3 Results

In two ALS patients, testing was unsuccessful for different reasons. In the first patient (ALSFRS-R: 19), severe generalized neurogenic muscle atrophy resulted in the absence of detectable visual muscle activity, evoked CMAP, and Doppler signals following stimulation. In the second patient (ALSFRS-R: 40), severe bulbar symptoms with persistent coughing and difficulties in maintaining a stable supine position resulted in substantial movement artifacts, precluding reliable recording of the evoked CMAP and Doppler signals. Furthermore, several individuals discontinued the measurement protocol due to pain induced by electrical stimulation. This discomfort was most pronounced during the assessment of the rectus femoris muscle; consequently, this muscle group accounted for the majority of unsuccessful recordings (ALS group: 4 muscle recordings; control group: 14 muscle recordings), whereas fewer dropouts occurred during the evaluation of the other muscles (ALS group: up to 2 muscle recordings; control group: up to 6 muscle recordings). The specific case numbers for each examined muscle are presented in Table 2.

**Table 2:** Descriptive statistics for DML and EMD parameters.

Muscle	ALS	Age-matched controls	Younger controls
Number of muscles examined			
ADM	50	54	50
BR	50	52	50
TA	52	52	50
RF	48	44	50
DML	Mean (SD/*IQR*) [95% CI]		
ADM	3.31 (0.6) [3.13–3.48]	2.91 (*1.0*) [2.74–3.07]	2.59 (0.4) [2.48–2.69]
BR	3.71 (*1.1*) [3.42–4.01]	3.57 (0.6) [3.40–3.73]	3.01 (*0.8*) [2.85–3.16]
TA	3.96 (*1.1*) [3.69–4.23]	3.22 (0.5) [3.09–3.35]	2.86 (*0.4*) [2.77–2.95]
RF	4.26 (*1.1*) [3.96–4.56]	3.76 (*0.8*) [3.53–3.99]	2.28 (0.4) [2.92–3.16]
EMD	Mean (SD/*IQR*) [95% CI]		
ADM	6.89 (2.6) [6.15–7.64]	4.80 (1.9) [4.29–5.33]	5.14 (1.8) [4.64–5.65]
BR	9.62 (3.9) [8.50–10.73]	6.46 (3.1) [5.60–7.32]	6.25 (2.7) [5.48–7.01]
TA	11.07 (2.8) [10.29–11.84]	6.29 (*4.1*) [5.63–6.94]	6.95 (2.4) [6.26–7.64]
RF	15.68 (*6.1*) [13.58–17.78]	10.45 (*5.4*) [8.49–12.42]	8.46 (3.15) [7.56–9.35]

The value in italics represents the interquartile range, which is applied in cases where the data group does not follow a normal distribution.

Exemplary measurement recordings for an ALS patient (No. 27) and an age-matched control subject (No. 15) across all four examined muscles are illustrated in Figure 4.

### 3.1 Compound muscle action potential amplitudes

In the ALS cohort, mean CMAP amplitudes ranged between 6.5 and 7.7 mV. In comparison, the age-matched control cohort exhibited mean amplitudes between 8.6 and 11.3 mV, while the younger control group showed values ranging from 10.9 to 20.5 mV (Supplementary Table 1, Supplementary Figure 1). Intergroup comparisons revealed significant differences across all examined muscles between the ALS cohort and both control groups, except for the rectus femoris muscle when comparing the ALS cohort to the age-matched controls.

### 3.2 Distal motor latency (DML)

The DML in ALS patients was prolonged compared to healthy controls (Table 2, Figure 5). Except for the comparison of the BR muscle between ALS patients and age-matched controls, all other deviations were statistically significant, with the majority of analyses conducted using the Mann–Whitney *U* test.

**Figure 5:**
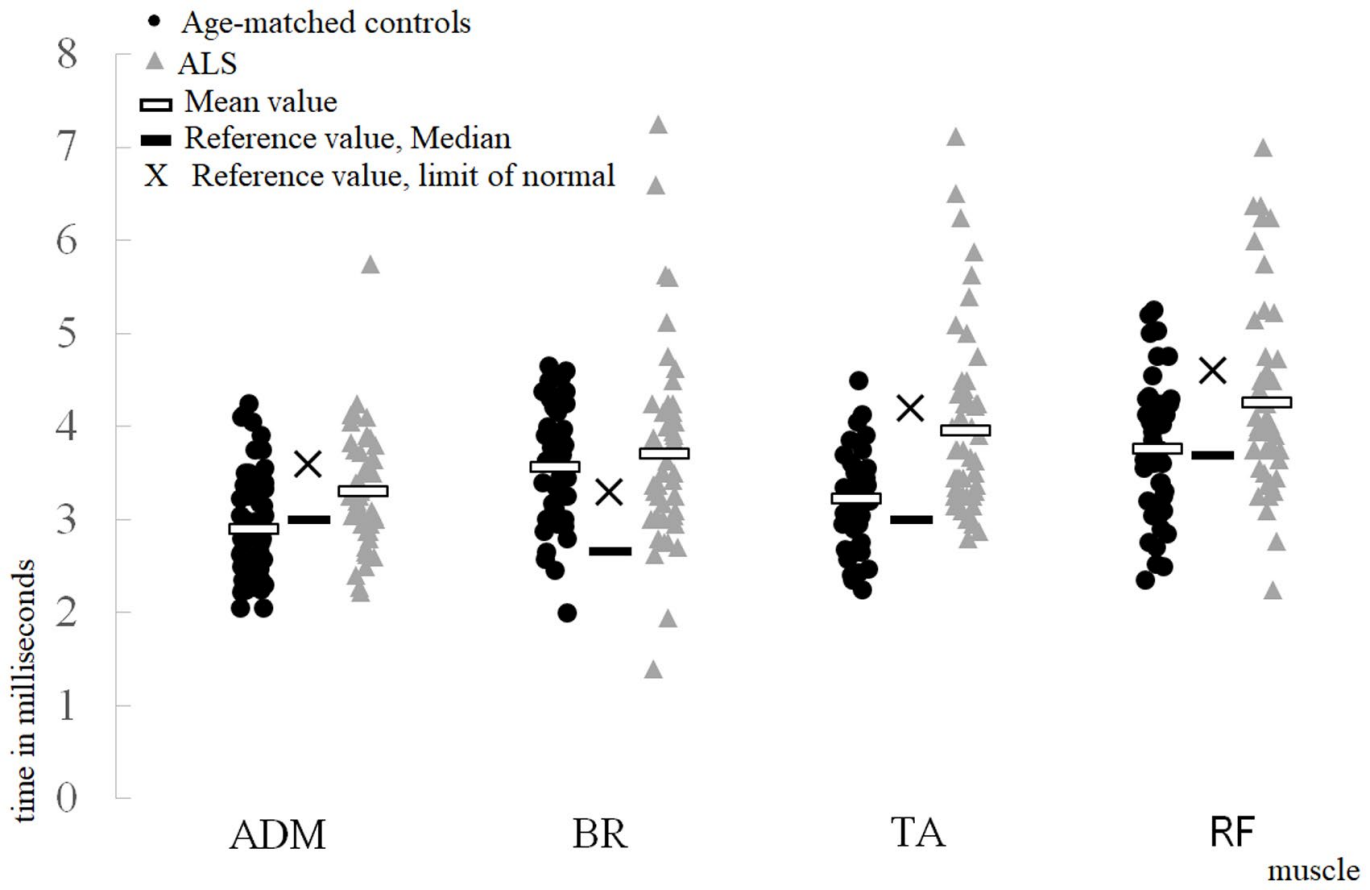
Distal motor latency of ALS patients and age-matched controls. The reference values for DML were extracted from common literature utilizing the same experimental setup (24–27).

When comparing the ALS cohort to age-matched controls, the greatest intergroup difference in mean values occurred in the TA muscle, amounting to 0.74 ms. In contrast, the BR muscle showed the least disparity, differing by only 0.14 ms on average. The DML times in the younger control group are generally shorter than those observed in the age-matched controls.

### 3.3 Correlation between distal motor latency and mechanical latency

The correlation between DML and mechanical latency (defined as the latency between electrical nerve stimulation and the initiation of the Doppler response) was analyzed to characterize the relationship between the electrophysiological CMAP and Doppler-derived measures and to determine the extent to which the two methods provide corresponding measures of response latency. A considerable part of interindividual variation in mechanical latency is not explained by variations in electrical latencies:

The correlation between DML and mechanical latency varied across muscle groups and study groups. In the ADM, a significant positive correlation was observed in all three groups, with small to moderate effect sizes (ALS: *r* = 0.287, *p* = 0.044; age-matched controls: *r* = 0.375, *p* = 0.005; younger controls: *r* = 0.418, *p* = 0.003). In contrast, for the RF, a significant correlation was observed only in the younger control group (*r* = 0.290, *p* = 0.041). Overall, significant correlations were observed in three muscle groups in both the ALS group (*r* ranging from 0.28 to 0.36) and the younger control group (*r* ranging from 0.29 to 0.42), whereas only one muscle group showed a significant correlation in the age-matched control group (*r* = 0.38). The observed correlations were generally small to moderate in magnitude. All correlations are depicted in Table 3.

**Table 3:** Correlation between distal motor latency and mechanical latency.

Muscle	ALS	Age-matched controls	Younger controls
ADM	***p* = 0.044; *r* = 0.287**	***p* = 0.005; *r* = 0.375**	***p* = 0.003; *r* = 0.418**
BR	***p* = 0.046; *r* = 0.284**	*p* = 0.408	*p* = 0.249
TA	***p* = 0.009; *r* = 0.359**	*p* = 0.077	***p* = 0.011; *r* = 0.360**
RF	*p* = 0.153	*p* = 0.879	***p* = 0.041; *r* = 0.290**

Associations between variables were evaluated using Spearman’s rank correlation coefficient. Bold values indicate statistical significance.

### 3.4 Electromechanical delay

Mean EMD values for the different muscle groups ranged from 6.9 to 15.7 ms for ALS patients, 4.8–10.5 ms for age-matched controls, and 5.1–8.5 ms for younger controls.

In direct group comparisons, ALS patients showed significantly prolonged EMD compared with healthy controls across all muscle groups (Figure 6, Table 2). The most pronounced effect is observed in the TA muscle, with a mean value of 11.07 ± 2.8 ms [10.29–11.84 ms] for ALS patients, compared to a mean of 6.29 ms (4.1 ms) [5.63–6.94 ms] for age-matched controls.

**Figure 6:**
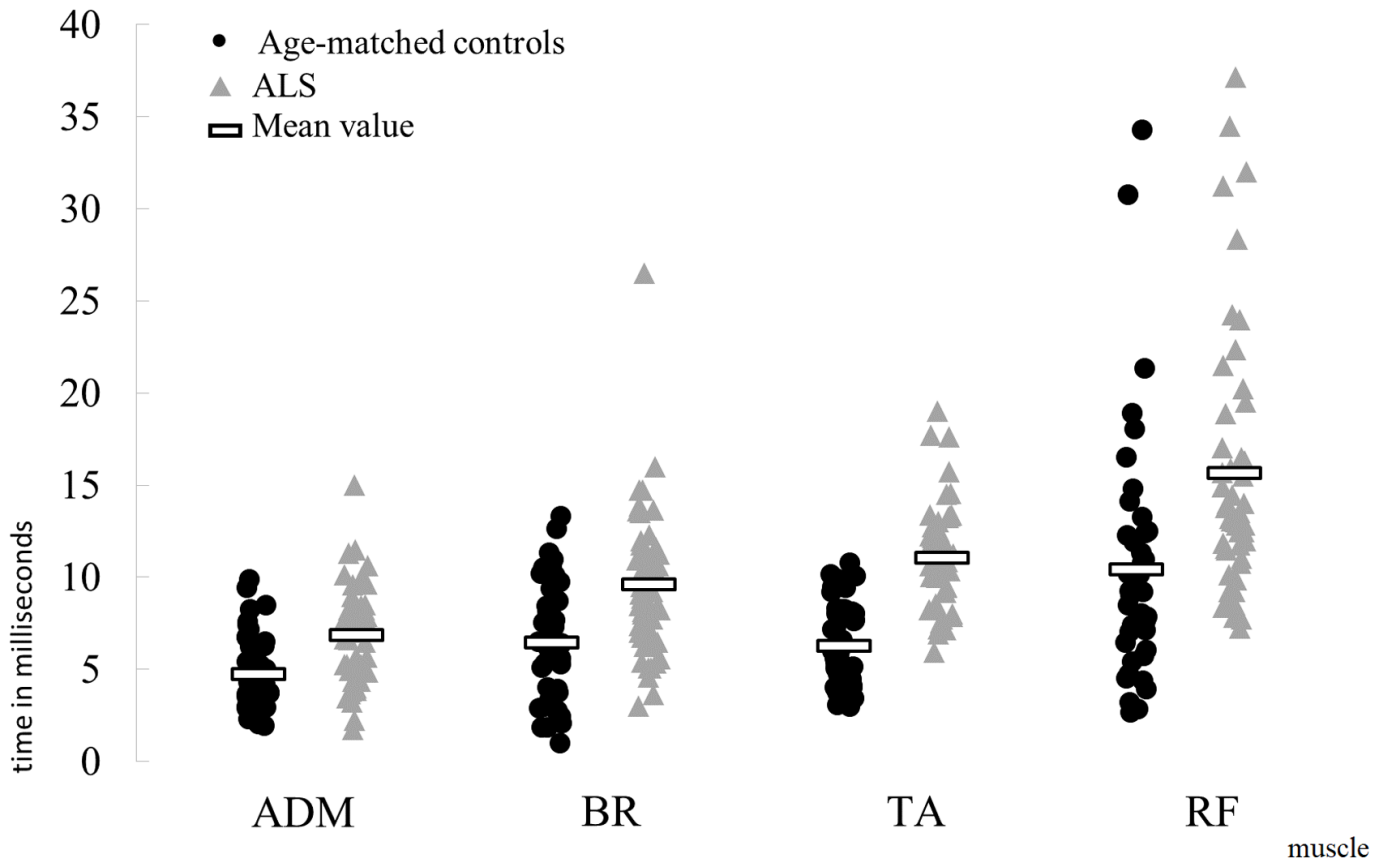
Electromechanical delay of ALS patients and age-matched controls.

To account for these repeated measurements in the statistical analysis, we used a linear mixed-effects model including group, age, sex, and muscle as fixed effects and patient as a random effect. Age did not significantly affect the outcome (*p* = 0.560), and no significant effect of sex was observed (*p* = 0.319). In contrast, a significant overall effect of group was identified (*p* < 0.0001). Post-hoc pairwise comparisons showed a significant difference between the ALS group and the age-matched control group (*p* < 0.0001). In contrast, the ALS group did not differ significantly from the younger control group (*p* = 0.210), and no significant difference was observed between the two control groups (*p* = 0.819).

A significant overall effect of muscle was also observed (*p* < 0.0001), indicating differences in the outcome across the four investigated muscles. *Post-hoc* pairwise comparisons revealed significant differences between ADM and BR, ADM and RF, ADM and TA, BR and RF, and RF and TA (all *p* < 0.0001). The comparison between BR and TA also reached statistical significance (*p* = 0.013). The complete results of the linear mixed-effects model analysis are presented in Table 4.

**Table 4:** Linear mixed model analysis.

Characteristics	*p*-value
Age	0.560
Sex	0.319
Group	<0.0001^a^
ALS vs. matched controls	<0.0001^b^
ALS vs. younger controls	0.210^b^
Matched controls vs. younger controls	0.819^b^
Muscles	<0.0001^a^
ADM vs. BR	<0.0001^b^
ADM vs. RF	<0.0001^b^
ADM vs. TA	<0.0001^b^
BR vs. RF	<0.0001^b^
BR vs. TA	0.0133^b^
RF vs. TA	<0.0001^b^

*p*-values of the linear mixed model log(EDM) ~ Group + Age + Sex + Muscle + (1|Patient).

a *p*-values for the overall comparison of differences between groups/muscles.

b Pairwise comparisons of the groups/muscles were performed *post-hoc* using a Wald test with Satterthwaite degrees of freedom, including a Tukey correction for multiple testing.

Through ROC analysis, the study assessed the performance of the EMD parameter, specifically its precision in classifying individuals as healthy or diseased. Table 5 presents the detailed results of the ROC analysis.

**Table 5:** ROC analysis of the electromechanical delay.

Muscle(s)	ALS and age-matched controls	ALS and younger controls
Investigation of individual muscles		
	**Sensitivity** [95% CI]/**1-Specificity** [95% CI] [**AUC** (95% CI)]	
ADM	**0.58** [0.442–0.706]/**0.167** [0.090–0.287] [**0.744** (0.648–0.839)]	**0.58** [0.442–0.706]/**0.200** [0.112–0.330] [**0.702** (0.599–0.804)]
BR	**0.84** [0.715–0.917]/**0.481** [0.351–0.613] [**0.742** (0.648–0.836)]	**0.56** [0.423–0.688]/**0.140** [0.070–0.262] [**0.774** (0.684–0.864)]
TA	**0.71** [0.577–0.817]/**0.058** [0.020–0.156] [**0.906** (0.852–0.959)]	**0.64** [0.504–0.757]/**0.060** [0.021–0.162] [**0.870** (0.805–0.936)]
RF	**0.75** [0.611–0.851]/**0.295** [0.180–0.442] [**0.764** (0.665–0.863)]	**0.79** [0.656–0.881]/**0.200** [0.112–0.330] [**0.853** (0.780–0.926)]
Investigation of muscle combinations		
	**Sensitivity** [95% CI]/**1-Specificity** [95% CI] [**AUC** (95% CI)]	
8 out of 8	**0.846** [0.665–0.938]/**0.222** [0.106–0.408] [**0.880** (0.789–0.971)]	
7 out of 8	**0.846** [0.665–0.938]/**0.222** [0.106–0.408] [**0.887** (0.799–0.976)]	
6 out of 8	**0.769** [0.579–0.890]/**0.111** [0.039–0.281] [**0.901** (0.817–0.985)]	
5 out of 8	**0.769** [0.579–0.890]/**0.037** [0.007–0.183] [**0.890** (0.798–0.983)]	
4 out of 8	**0.846** [0.665–0.938]/**0.074** [0.021–0.234] [**0.903** (0.816–0.990)]	
3 out of 8	**0.885** [0.710–0.960]/**0.185** [0.082–0.367] [**0.879** (0.781–0.976)]	
2 out of 8	**0.962** [0.811–0.993]/**0.259** [0.132–0.447] [**0.863** (0.756–0.969)]	

The highest discriminatory power for individual muscles was observed in the tibialis anterior (TA) muscle, with a Youden index of 0.654. When considering muscle combinations, the highest discriminatory power was achieved using a subset of 4 out of 8 muscles, yielding a Youden index of 0.732. For each model, the optimal diagnostic cut-off was determined using the highest Youden’s Index. Confidence intervals (95% CI) for sensitivity and false-positive rate were calculated using the Wilson score method. Bold values are used for clarity only.

When evaluating this question at the level of individual muscles, the TA exhibited the greatest discriminatory power in the ROC analysis, with an AUC of 0.906 and a Youden index of 0.654 for the ALS cohort and age-matched controls. Using an EMD cutoff of 10.0 ms for the TA muscle, ALS patients could be distinguished from age-matched healthy controls with a sensitivity of 71.2% and a specificity of 94.2%.

Additionally, an analysis was conducted to assess the potential enhancement of discriminatory ability through a combined evaluation of individual muscles. A reference scale was established using data from age-matched controls, considering percentiles and excluding outliers by employing a threshold set at 1.5 times the interquartile range from the first and third quartiles. Using this reference scale, ALS patients were also categorized. The evaluation involved testing and classifying combinations of the two to eight largest EMD values obtained from the eight examined muscles for each individual.

The greatest AUC was observed when four out of the eight muscles were selected. Although the AUC was slightly lower at 0.903 compared to the isolated consideration of the TA, the Youden Index showed an increase of 0.078 points. At the cutoff value corresponding to the highest Youden Index, a sensitivity of 84.6% was achieved with a specificity of 92.6%, indicating that discriminative capability was enhanced through the examination of muscle combinations as opposed to individual assessments. Given that the muscle combination approach prioritized muscles with the highest EMD values, the TA was frequently included among the selected muscles and consequently had a substantial influence on the overall score in the combined muscle assessment. Beyond the TA, no consistent pattern supporting a fixed muscle combination emerged. In several analyses, the optimal combinations included unilateral measurements from different muscles rather than consistently selecting the same muscle bilaterally. The graphical ROC analyses are presented in Figures 7, 8.

**Figure 7:**
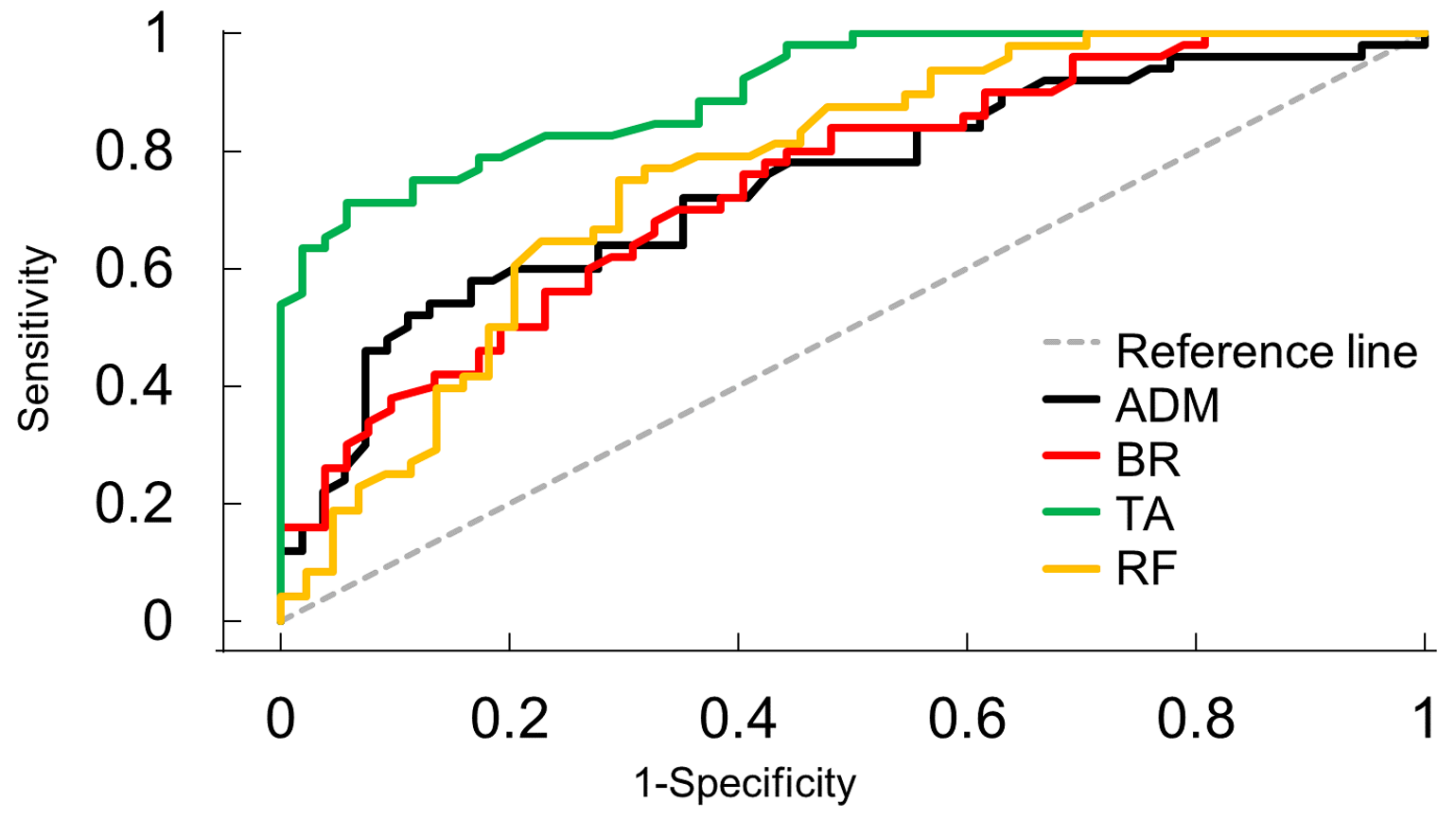
ROC analysis of EMD in ALS patients and age-matched controls. Muscle-specific analysis.

**Figure 8:**
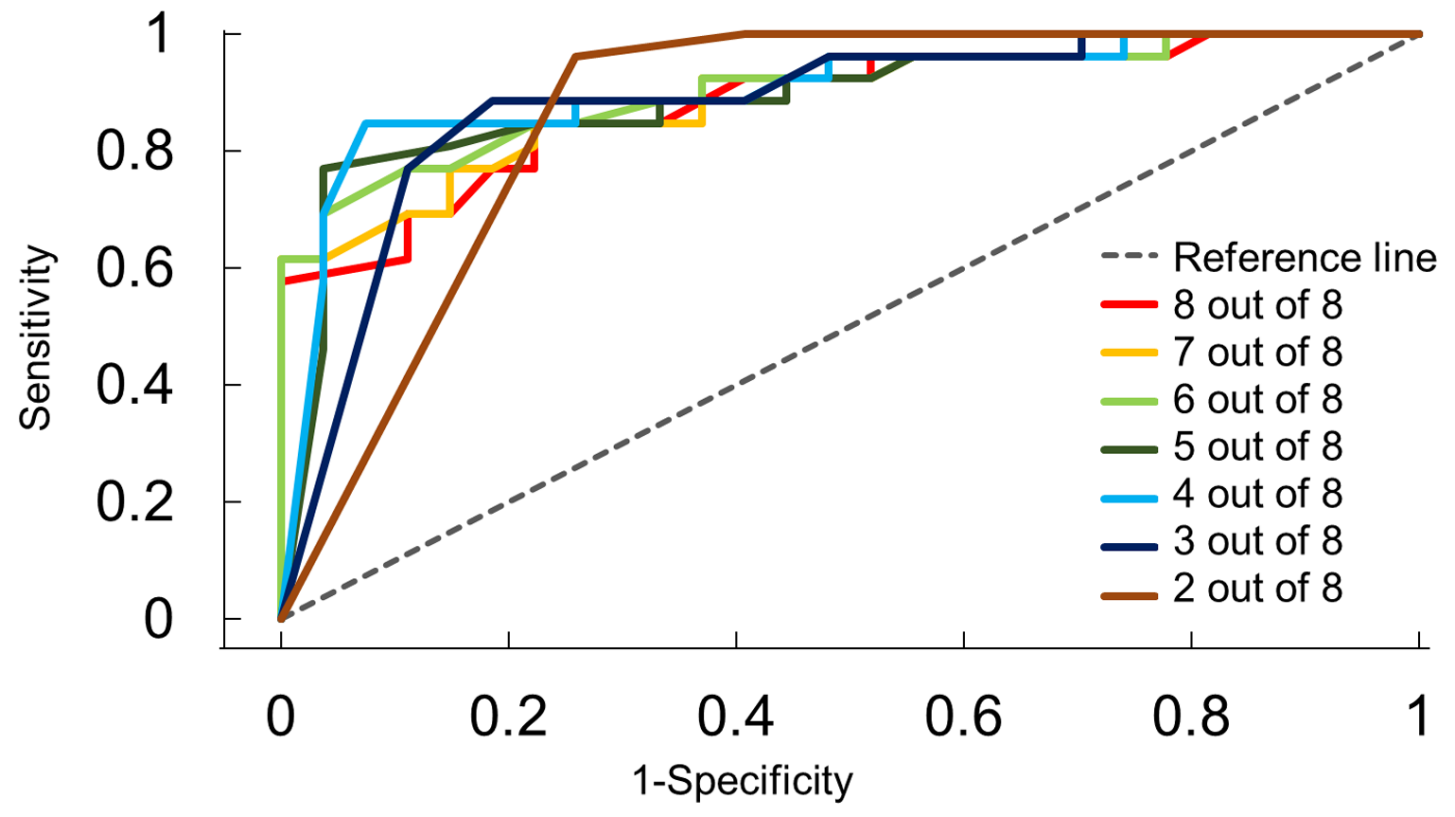
ROC analysis of EMD in ALS patients and age-matched controls. Analysis of different muscle combinations. For the selection of evaluated muscles, those with the longest delays were considered.

No significant differences in EMD were observed between the primary lateral sclerosis subgroup and the rest of the ALS cohort. Across the combined muscle evaluation, *Z*-score deviations remained within ± 0.8, indicating substantial overlap in the measured values. Furthermore, no significant correlations were found between EMD measurements and the clinical data collected from ALS patients (*p* > 0.05 for all comparisons). Specifically, no associations were observed between EMD in the TA muscle and Achilles tendon reflex strength, trophic condition of the legs, severity of fasciculations, cramp index score (23), ALSFRS-R score, or disease duration.

### 3.5 Association between EMD and CMAP amplitude in the ALS cohort

To evaluate the association between EMD and CMAP amplitude within the ALS cohort, muscle-specific simple linear regression analyses were performed, with CMAP amplitude as the independent variable and EMD as the dependent variable. Statistically significant regression models were observed for the RF (*R*^2^ = 0.290, *p* < 0.001) and BR (*R*^2^ = 0.1363, *p* = 0.0083), whereas the models for the ADM (*R*^2^ = 0.0737, *p* = 0.0592) and TA (*R*^2^ = 0.0128, *p* = 0.424) did not reach statistical significance.

## 4 Discussion

This study presents the results of a direct comparison between electrical and mechanical measures of neuromuscular transmission in ALS patients and healthy controls, obtained using a novel sonoelectromyography system. We demonstrate a marked increase of EMD in ALS patients compared to controls, suggesting impaired excitation–contraction coupling. These findings provide evidence of altered electromechanical muscle properties in ALS and offer further insight into changes in muscle contractility associated with the disease.

To define normative and threshold values for DML, relevant studies with comparable measurement conditions were utilised as references. The DML reference values reported in the literature were 3.0 ms for the ADM (24), 2.66 ms for the BR (25), 3.0 ms for the TA (26), and 3.7 ms for the RF (27). The DML values in the control groups were generally in accordance with these reference values. In the age-matched control group, the DML values for ADM, TA, and RF deviated from the respective reference values by less than 10% (ADM: −3.0%; TA: +7.3%; RF: +1.6%). The BR showed a greater deviation, with a DML that was 0.91 ms longer than the reference value (+34.2%). In the younger control group, the BR likewise showed a slightly prolonged DML (+0.35 ms; +13.2%), whereas the DML values for ADM, TA, and RF were lower than the respective reference values (−13.7%, −4.7%, and −17.8%, respectively).

Several factors may account for the deviations from previously reported DML reference values, including differences in sample sizes and age between the control cohorts. Notably, the younger control group generally showed shorter DML values than the age-matched control group. This finding is consistent with previous studies reporting longer distal motor latencies with increasing age (28).

The deviations in DML values between ALS patients and control individuals in this study are at a disease-typical level. Prolonged DML values are typically observed at a moderate level of ≤125% in ALS (29). The greatest extension observed in this study was 116% for the TA, indicating that the examined cohort can be considered ALS-typical. Several pathophysiological mechanisms may contribute to these mild DML prolongations. These may include the loss of large, fast-conducting motor axons (30), alterations in axonal membrane excitability (31), and distal axonal changes (32) associated with motor unit degeneration and denervation–reinnervation processes. Importantly, the relatively mild prolongation of DML observed in ALS differs from the marked conduction slowing typically associated with demyelinating neuropathies.

The analysis of the correlation between DML and mechanical latency requires further consideration. Although significant correlations were observed in some muscle groups and study cohorts, the corresponding effect sizes were generally small to moderate. A certain degree of correlation between DML and mechanical latency is expected, as DML constitutes part of the overall latency between electrical nerve stimulation and the onset of the mechanical response. Nevertheless, the relatively limited strength of the observed associations supports the notion that EMD provides complementary information to conventional DML assessment rather than merely reflecting distal motor conduction latency.

Interestingly, the strength and consistency of the association differed between the age-matched and younger control groups. One possible explanation for the relatively weak correlations is the greater interindividual variability in mechanical latency compared with DML. Notably, mechanical latency exhibited greater interindividual variability in the age-matched control group than in the younger controls, which may have contributed to the weaker and less consistent correlations observed in this subgroup.

From a physiological perspective, this distinction is plausible, as EMD reflects the temporal sequence of biological processes occurring following electrical activation of the sarcolemma, including calcium release, calcium binding to troponin, initiation of the cross-bridge cycle, and subsequent biomechanical changes within the muscle and surrounding connective tissue.

The linear mixed-effects model demonstrated a significant overall group effect after accounting for age, sex, and muscle. *Post hoc* analyses revealed a highly significant difference between ALS patients and age-matched controls, whereas the comparison between ALS patients and younger controls did not reach statistical significance. Notably, age itself was not a significant fixed effect in the model, suggesting that the absence of a significant difference between ALS patients and younger controls cannot be readily attributed to an independent effect of age. Instead, differences in within-group variability, sample characteristics, and statistical power may have contributed to the differing results of the pairwise comparisons. Thus, the present findings provide strongest evidence for prolonged EMD in ALS when compared with age-matched healthy controls.

The greatest deviation was observed in the TA muscle. This finding may indicate either a greater suitability of the TA for detecting alterations in EMD or an earlier or more pronounced disease-related involvement compared with the other muscles examined. In the ROC analyses, the TA was also the muscle most frequently included among the combinations yielding the highest scores. However, beyond the TA, no clear evidence for a fixed muscle combination emerged. In several analyses, the highest scores were obtained using unilateral measurements from different muscles rather than consistently selecting the same muscle bilaterally. Therefore, while the TA may be particularly suitable for EMD assessment in ALS, the present data do not support the recommendation of a standardized muscle combination. Future studies should investigate the extent to which EMD prolongation is associated with the degree of atrophy of the respective muscle, which may help to clarify whether the pronounced deviation observed in the TA is related to muscle atrophy or reflects other disease-related mechanisms. A characteristic pattern of muscle involvement has been described in ALS, with preferential weakness of specific muscle groups even in the early stages of the disease, despite a clinically focal onset (33). The so-called “split” phenomena have been described in both the upper and lower extremities and have been associated with differences in corticomotoneuronal innervation. In the lower extremities, preferential involvement of the ankle dorsiflexors compared with the plantar flexors has been reported (34), which may contribute to the particular suitability of the TA for detecting disease-related EMD alterations in our study.

The prolongation of the EMD documented here aligns with earlier reported findings, implying impaired electromechanical coupling in ALS patients (4, 35). However, these prior investigations relied on fundamentally different methodologies with distinct limitations. Schmied et al. (4) evaluated coupling efficiency mathematically in a small cohort of eleven ALS patients by calculating the ratio between the electrical macro-motor unit potential (macro-MUP) area and the mechanical twitch contraction force. This mechanical component was recorded via a force transducer at the wrist, thereby capturing indirect force transmission across joints and tendons during voluntary contraction rather than isolating the muscle belly itself. More recently, Planinc et al. (35) investigated a sample of twelve ALS patients and focused exclusively on spontaneous fasciculations. Their setup combined high-density surface electromyography (HD-sEMG) to map local electrical activity with B-mode muscle ultrasound videos. The mechanical event was subsequently identified using mathematically intensive pixel-shifting algorithms to track passive tissue displacement. In contrast to previous investigations, the CMAP and Doppler signals in the present setup were recorded at identical sampling frequencies and from the exact same anatomical site, enabling a highly precise determination of the temporal intervals between these two events.

Current research into ALS has increasingly focused on the neuromuscular junction (NMJ) and primary muscle pathology as critical sites of early disease onset (36, 37). According to the “dying-back” hypothesis, pathological alterations within skeletal muscle, together with degeneration of the NMJ and distal axons, may precede the loss of motor neurons in the central nervous system (38, 39). In this context, disturbances in L-type calcium channels (Ca_V_1.1), acting as voltage sensors, have been identified in ALS animal models. This dysfunction impairs ryanodine receptor activation, reducing sarcoplasmic calcium release and delaying muscle contraction (40). Such alterations indicate substantial impairments in excitation–contraction coupling and neuromuscular signal transmission. Impaired sarcolemmal repair mechanisms may further exacerbate these alterations, compromising muscle membrane integrity. These defects, largely driven by dysregulation of membrane repair proteins such as MG53, promote increased sarcolemmal fragility and elevate oxidative stress within the myofiber (41).

Beyond peripheral muscular alterations, motor neuron degeneration drives a progressive remodeling of the neuromuscular network. This phenomenon initiates motor unit reorganization and compensatory collateral sprouting by surviving motor neurons (42), which may temporarily mask ongoing NMJ dismantlement (39). Furthermore, this structural rearrangement is closely linked to enhanced spinal network excitability (43). Crucially, these reorganized motor units exhibit altered synchronization patterns and reduced functional efficiency (4, 8). In addition to these neural impairments, spastic muscles undergo extensive structural remodeling. These changes include modifications in muscle fiber size and composition, an increased accumulation of extracellular matrix and connective tissue, greater passive stiffness of muscle fibers, and compromised mechanical performance of the extracellular matrix. Collectively, these structural changes modify the biomechanical properties of the muscle and contribute to the functional deficits observed in spasticity (44).

EMD measurements could improve the diagnosis and disease monitoring of ALS in clinical practice by providing an objective, bedside estimate of the extent of peripheral muscle alterations. According to the current Gold-Coast criteria, lower motor neuron dysfunction can be documented either by demonstrating muscle weakness and muscle wasting during the clinical examination or by utilizing conventional EMG. Within the electrodiagnostic framework, the consensus criteria strictly require the concurrent evidence of chronic neurogenic remodeling, as well as signs of ongoing denervation (45). However, specific measurement methods designed to quantify these early pathological alterations are not yet integrated into these diagnostic criteria. Therefore, the measurement of EMD using our novel methodology may provide complementary information on early neuromuscular alterations and could potentially contribute to the detection of lower motor neuron dysfunction at an earlier stage. Of particular significance would be the implementation of this methodology for the assessment of facial or bulbar muscles, potentially serving as a non-invasive and more comfortable alternative to conventional needle electromyography.

The present study has several limitations that warrant consideration. The proposed cut-off values should be validated in larger, more homogeneous cohorts, particularly in patients assessed at earlier stages of the disease. The diagnostic specificity of these cut-off values remains to be established, as they were derived exclusively from comparisons with healthy controls in the present study. Future studies should therefore include appropriate disease control groups, such as patients with ALS mimic disorders, particularly multifocal motor neuropathy, to determine whether EMD alterations can specifically distinguish ALS from other neuromuscular disorders. Furthermore, although all measurements were performed by a single investigator (SW), thereby eliminating interrater variability, validation in larger and more diverse cohorts is required. Larger multicenter studies should ideally use optimized experimental protocols, including strict control of skin temperature. In addition, potential confounding factors that are difficult to quantify, such as the biomechanical properties of skeletal muscle and the passive musculoskeletal system, should be considered. Rigorous standardization and control of limb positioning and movement throughout testing will be essential to further improve measurement accuracy and reproducibility.

## Data Availability

The raw data supporting the conclusions of this article will be made available by the authors, without undue reservation.
