# Supplementary material for: Simultaneous evoked CMAP recording and sonography reveal a prolonged electromechanical delay in ALS patients
Source: Front Neurol. 2026 Oct 28;17:1943566. doi: 10.3389/fneur.2026.1943566 (PMC13751537; doi:10.3389/fneur.2026.1943566)
Supplement: Supplementary file 1 [file Data_Sheet_1.pdf]

Supplementary Material

Supplementary Table 1 Descriptive statistics for CMAP amplitude.

	ALS	Age-matched controls	Younger controls
**CMAP amplitude**	Mean (SD/*IQR*) [95% CI]		
ADM	6.53 (3.61) [5.49 - 7.57]	8.61 (2.74) [7.86 - 9.36]	10.85 (*3.26*) [9.98 – 11.72]
BR	7.68 (3.30) [6.74 - 8.62]	9.38 (3.64) [8.35 - 10.40]	12.22 (3.48) [11.23 – 13.21]
TA	7.07 (4.02) [5.95 - 8.18]	11.26 (3.39) [10.32 - 12.21]	13.63 (*3.58*) [12.72 – 14.54]
RF	7.12 (4.88) [5.67 - 8.57]	8.76 (4.70) [7.25 - 10.26]	20.51 (4.34) [19.28 – 21.74]

in mV

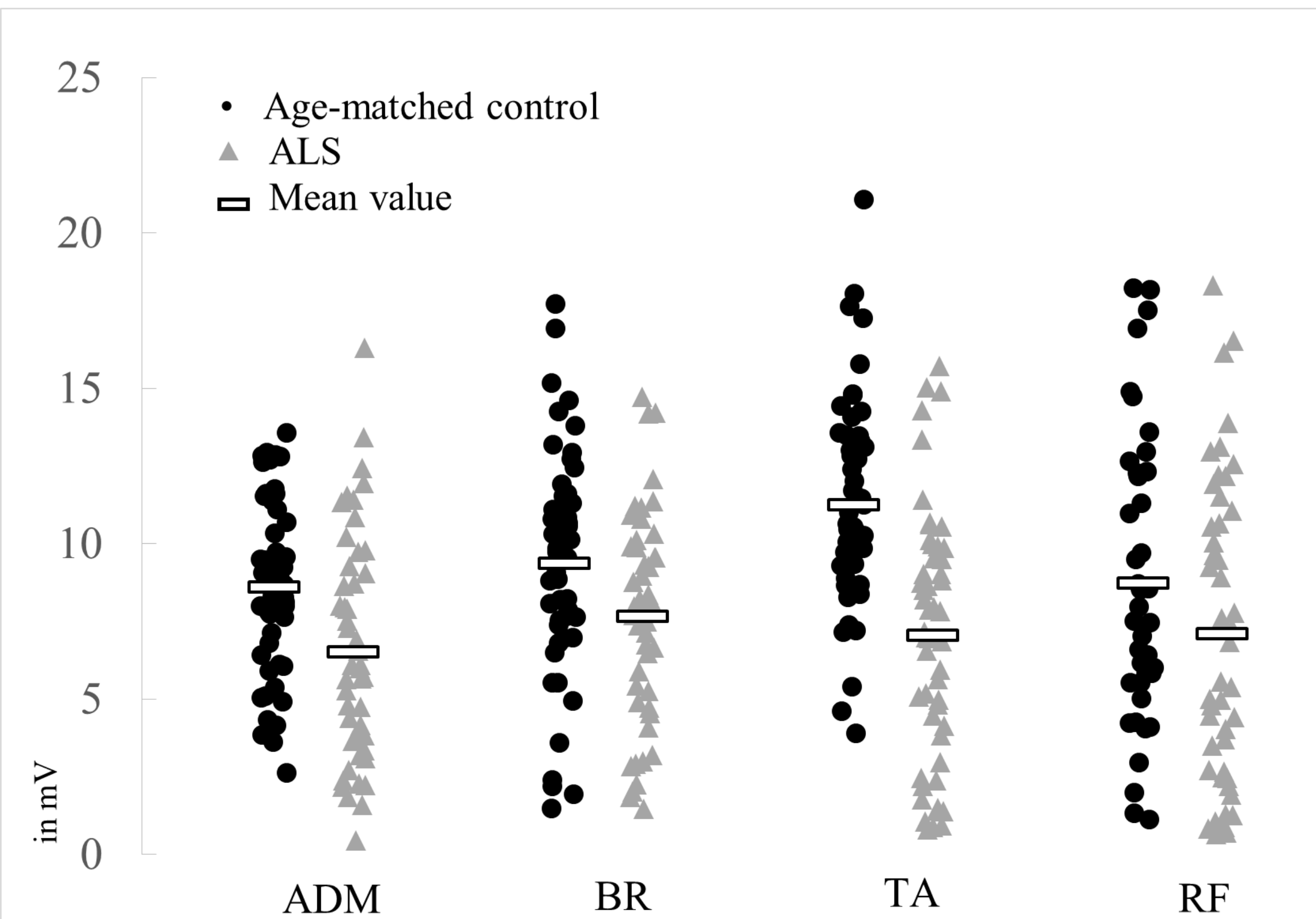

Supplementary Fig. 1 CMAP amplitude of ALS patients and age-matched controls.
